# Neonatal Resuscitation With T-Piece Systems: Risk of Inadvertent PEEP Related to Mechanical Properties

**DOI:** 10.3389/fped.2021.663249

**Published:** 2021-06-07

**Authors:** Thomas Drevhammar, Markus Falk, Snorri Donaldsson, Mark Tracy, Murray Hinder

**Affiliations:** ^1^Anaesthesiology and Intensive Care Medicine, Department of Surgical and Perioperative Sciences, Umeå University, Umeå, Sweden; ^2^Department of Women's and Children's Health, Karolinska Institute, Stockholm, Sweden; ^3^Neonatology Department, Karolinska University Hospital, Stockholm, Sweden; ^4^Department of Pediatrics and Child Health, The University of Sydney, Sydney, NSW, Australia; ^5^Neonatal Intensive Care Unit, Westmead Hospital, Sydney, NSW, Australia

**Keywords:** resuscitation, infant, newborn, positive pressure ventilation, equipment design, expiratory time constant, resistance to breathing, inadvertent PEEP

## Abstract

**Background:** Resuscitation of infants using T-piece resuscitators (TPR) allow positive pressure ventilation with positive end-expiratory pressure (PEEP). The adjustable PEEP valve adds resistance to expiration and could contribute to inadvertent PEEP. The study indirectly investigated risk of inadvertent peep by determining expiratory time constants. The aim was to measure system expiratory time constants for a TPR device in a passive mechanical model with infant lung properties.

**Methods:** We used adiabatic bottles to generate four levels of compliance (0.5–3.4 mL/cm H_2_O). Expiratory time constants were recorded for combinations of fresh gas flow (8, 10, 15 L/min), PEEP (5, 8, 10 cm H_2_O), airway resistance (50, 200 cm H_2_O/L/sec and none), endotracheal tube (none, size 2.5, 3.0, 3.5) with a peak inflation pressure of 15 cm H_2_O above PEEP.

**Results:** Low compliances resulted in time constants below 0.17 s contrasting to higher compliances where the expiratory time constants were 0.25–0.81 s. Time constants increased with increased resistance, lower fresh gas flows, higher set PEEP levels and with an added airway resistance or endotracheal tube.

**Conclusions:** The risk of inadvertent PEEP increases with a shorter time for expiration in combination with a higher compliance or resistance. The TPR resistance can be reduced by increasing the fresh gas flow or reducing PEEP. The expiratory time constants indicate that this may be clinically important. The risk of inadvertent PEEP would be highest in intubated term infants with highly compliant lungs. These results are useful for interpreting clinical events and recordings.

## Introduction

T-piece resuscitators (TPR) are used for resuscitation of newborn infants worldwide and recommended in international guidelines (1). These devices allow positive pressure ventilation (PPV) with positive end expiratory pressure (PEEP) of infants with inadequate respiratory effort and continuous positive airway pressure treatment (CPAP) of breathing infants.

The PEEP and CPAP of TPR systems are generated by a fresh gas flow leaving the breathing circuit through an adjustable expiratory resistance valve. The same principle was used by Gregory et al. when CPAP was first used for newborn infants in respiratory distress (2). The adjustable resistor valve generates PEEP by interposing expiratory resistance with the un-intended effect of prolonging the time required for the lungs to deflate. In previous studies on TPR systems, a risk of inadvertent PEEP, also referred to as auto-PEEP, was identified (3, 4).

Several factors determine incomplete expiration, air trapping and inadvertent PEEP. The lung mechanics have been well-described and can be found in standard textbooks on mechanical ventilation (5). In adults, the most common reasons are increased airway or endotracheal tube resistance in combination with insufficient time for expiration (6–8). Adult ventilators allow for measurements of intrinsic PEEP using an end expiratory pause, after which the expiratory time constants can be obtained from the flow-volume curve (6, 7, 9, 10). As far as we know, there is no equivalent technology available when providing PPV using a TPR to ventilate infants.

Standard lung mechanics during passive expiration explain the risk for incomplete expiration in an infant would be higher with an increased airway resistance, the use of narrow diameter endotracheal tubes and with higher ventilatory rates in combination with high compliances and larger tidal volumes (5, 7, 8).

The overall aim of this study was to provide a basic mechanical understanding of expiration through a TPR of newborn infants. The used model also included compliances higher than in newborn infants but within the weitght limits of TPR systems (<10 kgs). We hypothesized that a risk of inadvertent PEEP due to incomplete expiration could be identified in simulations with high compliances, high airway or endotracheal tube resistances and high TPR resistances related to low fresh gas flows and high PEEPs. The study indirectly investigated risk of inadvertent peep by determining expiratory time constants. The aim was to measure system expiratory time constants for a TPR device in a passive mechanical model with infant lung properties.

## Materials and Methods

The study did not involve humans or animals and did not require an ethical board review. The expiratory time constants measured referred to time constants for deflations of the total system. Expiration or deflation was used to describe flow from the lung model.

Three adiabatic bottles were combined to give fixed compliances of 0.5, 1.1, 2.2, and 3.4 mL/cm H_2_O. The stiff bottles filled with metal wool were connected using short tubing and wide bore Y-connectors. Compliance was measured as tidal volume divided by inflation pressure (data from all experiments available in Supplementary Table 1a). Flow and pressure were measured at the lung model connection. The adiabatic bottles give a linear compliance at gas compression, and to this model, ET-tubes, standardized airway resistors and TPRs can be added. A set of experiments without using a TPR was performed to validate the method and for comparisons when reporting effects of expiration through the TPR (available in Supplementary Table 1b).

The Neopuff TPR driver (Fisher & Paykel, New Zealand) was used with the Neopuff dedicated T-piece (with suction port). The flexible, corrugated tubing from the driver to the T-piece was replaced with stiff tubing with the same internal diameter to minimize artifacts from fluctuations in fresh gas flow related to the corrugated tube compliance.

Two sets of experiments were conducted. The first set was conducted to investigate the *effects of fresh gas flow and PEEP level*. The experiments were conducted with a Rp50 airway resistor (Michigan Instruments, Michigan, USA) and without endotracheal tubes. Dry fresh gas flows of 8, 10, and 15 L/min were used in combination with a PEEP of 5, 8, or 10 cm H_2_O. The peak inflation pressure (PIP) was adjusted to 15 cm H_2_O above PEEP to keep tidal volumes constant at each level of compliance.

The second set of experiments was conducted to investigate the *effects of airway and endotracheal tube resistances*. The experiments were conducted with a fresh gas flow of 10 L/min and 5 cm H_2_O PEEP. The used airway resistances were Rp50 or Rp200 (Michigan Instruments, Michigan, USA) and the endotracheal tubes were uncut with sizes of 2.5, 3.0, or 3.5 mm internal diameter (blue line, Portex, Smiths Medical, Minneapolis, Minnesota, USA). For investigations on the effects of airway and endotracheal tube resistance, TPR settings were limited to a fresh gas flow of 10 L/min and 5 cm H_2_O PEEP using a PIP of 15 cm H_2_O above PEEP.

A minimum of 11 test lung deflations were collected at a rate, allowing sufficient time for each inflation and deflation cycle to be complete. Data was collected using the Spectra data acquisition software (v. 3.0.1.3, Grove Medical, UK). Flow and pressure were measured using a Florian (ACUTRONIC Medical Systems AG Hirzel, Switzerland). A traceable reference ventilator analyzer (FP-300, IMT Analytics AG, Switzerland) was used to measure fresh gas flow and calibrate the Florian at 10 L/min (checked for symmetrical performance and confirmed with a fixed volume syringe) and the pressure was calibrated at 0 and 30 cm H_2_O.

The Spectra software output variables for each passive expiration included the time constant (from flow-volume loops), compliance, pressure and tidal volume. Data from 10 deflations (number 2–11) were analyzed in SPSS (v. 26, IBM, Armonk, New York, USA). The used arbitrary reference of 0.17 s expiratory time constant was calculated based on an expiratory time of 0.5 s and the three time constants needed to complete expiration. The calculated highest inflation rate that ensured adequate expiration (referred to as highest safe ventilatory rate) was a theoretical value obtained from a fixed inflation time of 0.5 s before adding three time constants. Means were compared using ANOVA with Bonferroni correction for multiple comparisons. Statistical significance was set to *p* < 0.05.

## Results

**Figure 1:**
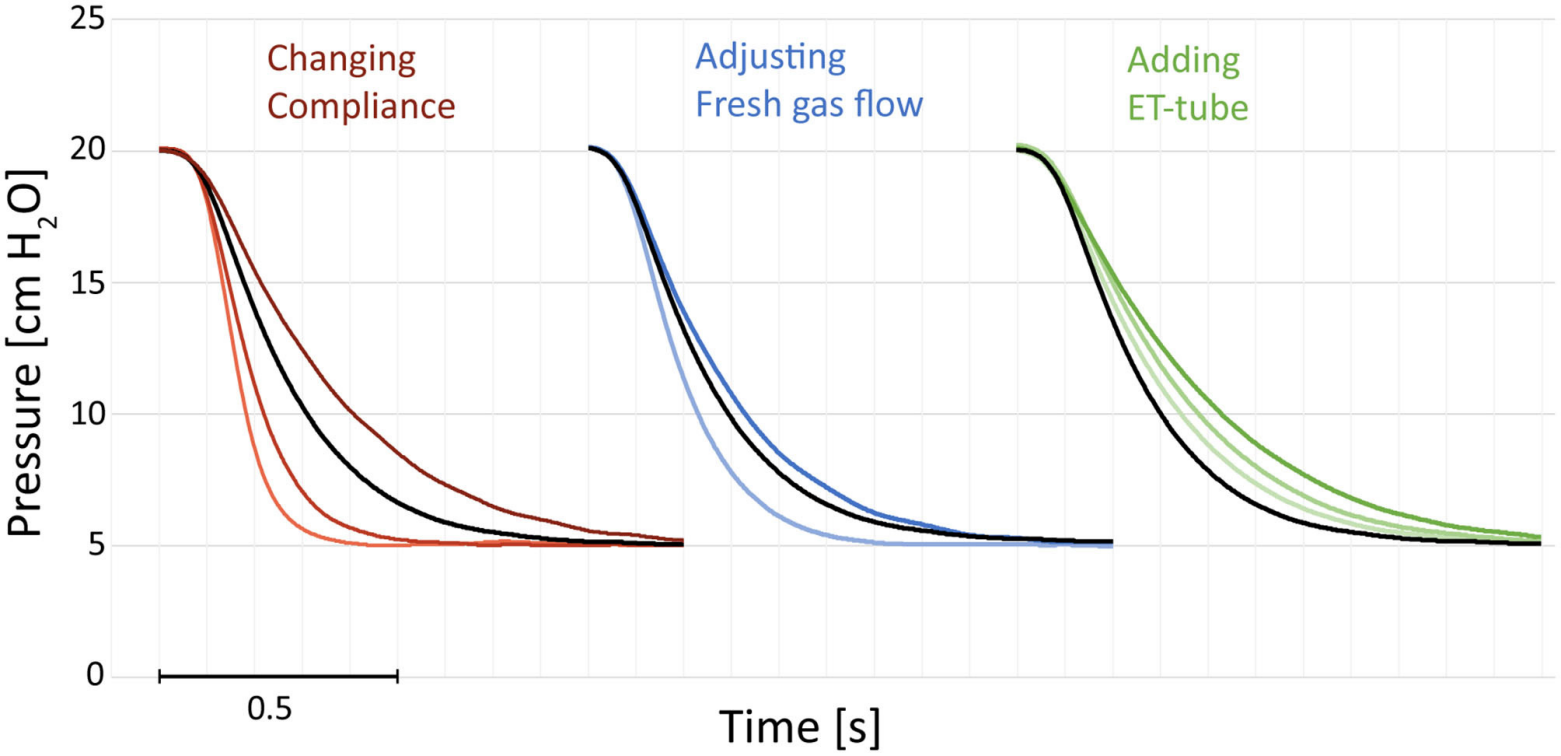
Examples of pressure recordings of single expirations. The figures illustrate the effect of compliance, resistance added by a change in fresh gas flow or adding an endotracheal (ET) tube. Increased compliance (darker red) increases the expiratory time. Decreasing the fresh gas flow (darker blue) increases the expiratory time. Adding an ET-tube increases the expiratory time (green). Black reference recorded with compliance 2.2 mL/cm, no ET-tube, and 10 L/min fresh gas flow. All recordings at 5 cm H_2_O and an airway resistor Rp50 with 20 cm H_2_O of peak inspiratory pressure. The single expirations have been graphically adjusted in time (X-axis) and were recorded separately.

The expiratory time constants increased with larger tidal volumes (resulting from inflation pressure and compliance) and higher system resistances. Examples of expirations illustrate increased time constants with prolonged time needed for a reduction in pressure (Figure 1).

### Compliance

**Figure 2:**
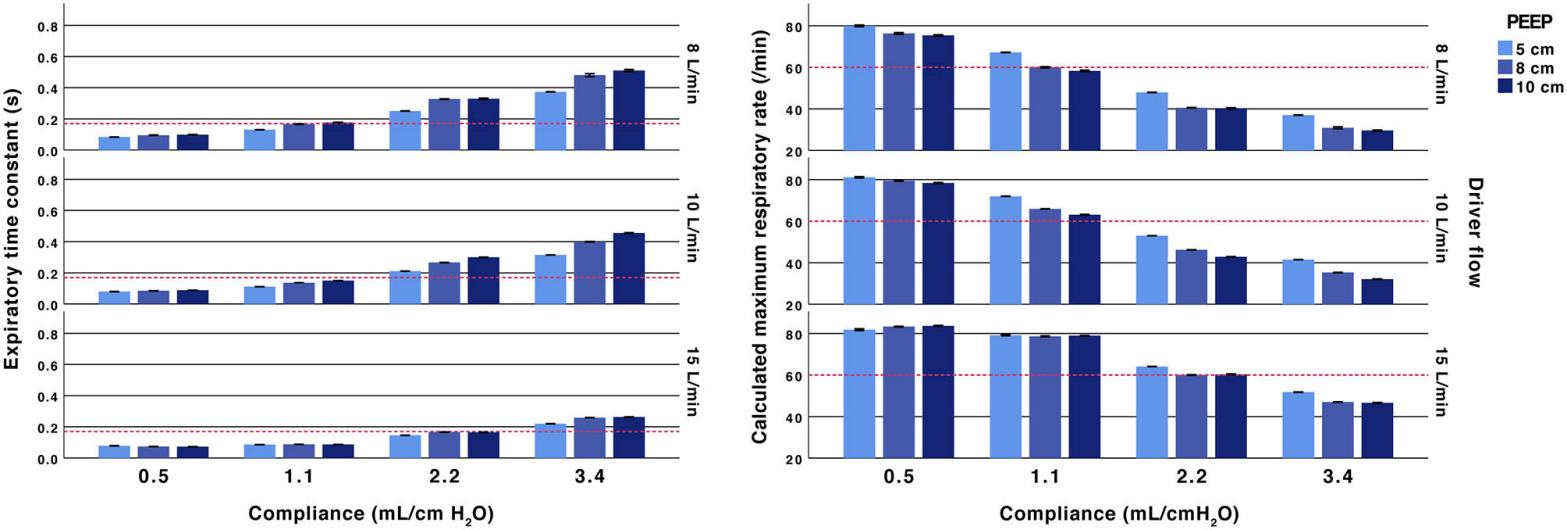
Effect of fresh gas flow, PEEP and compliance. Expiratory time constants (left) and calculated maximum ventilatory rate (right). All test performed with an inspiratory pressure of 15 cm H_2_O above PEEP and with an Rp50 airway resistor. Arbitrary reference lines at τrs 0.17 s represent a complete expiration time of 0.5 s (3*τrs) and a calculated maximum ventilatory rate of 60/min (fixed inflation time of 0.5 s). The expiratory time constant increases with higher compliance, lower fresh gas flow and a higher PEEP.

**Figure 3:**
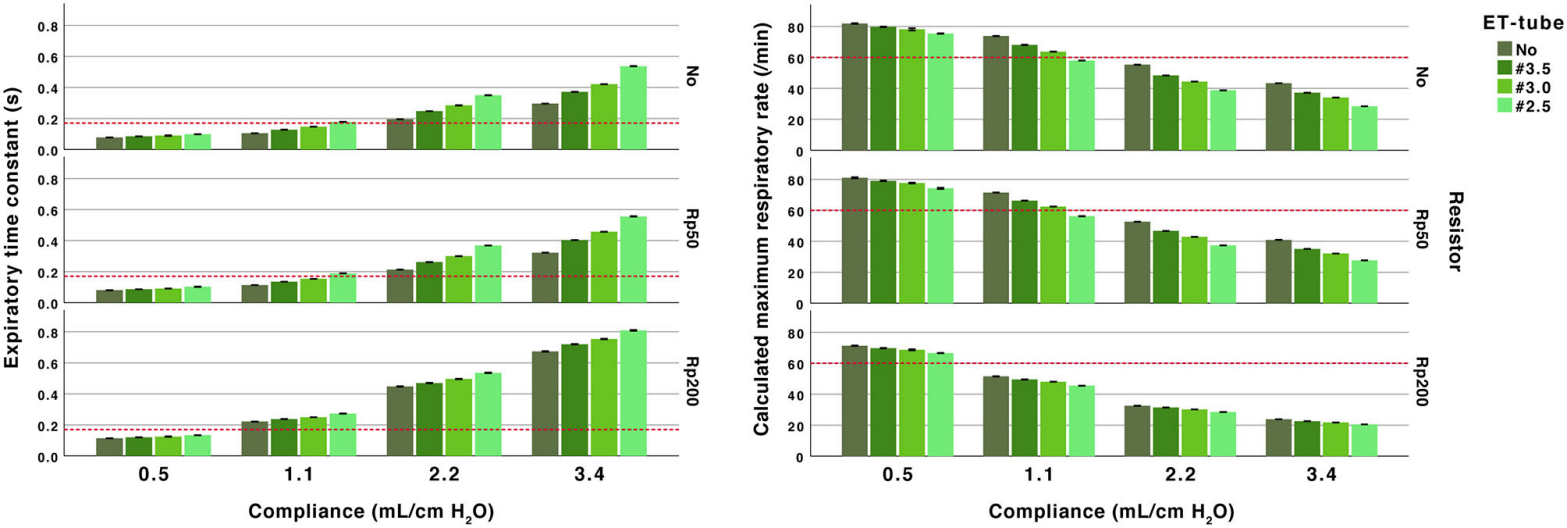
Effect of airway resistance, endotracheal (ET) tube and compliance. Expiratory time constant (left) and calculated maximum ventilatory rate (right). All performed at PEEP 5 and peak inspiratory pressure of 20 cm H_2_O at 10 L/min fresh gas flow. Arbitrary reference lines at τrs 0.17 s represent a complete expiration time of 0.5 s (3*τrs) and at a calculated maximum ventilatory rate of 60/min (fixed inflation time of 0.5 s). The expiratory time constant increase with higher compliance and higher resistance (ET-tube, airway resistor or both).

There was an increase in time constants with increased compliances and tidal volumes (Figures 1–3, Supplementary Tables 1–3) in all simulations.

The lung models with low compliances, 0.5 and 1.1 mL/cm H_2_O, had short time constants. For the low compliance experiments, all recordings with compliances of 0.5 mL/cm H_2_O the time constants were below 0.17 s, corresponding to a theoretical safe ventilatory rate of at least 60 inflations/min. With a compliance of 1.1 mL/cm H_2_O the time constants were above 0.17 s when high resistances were used (2.5 ET-tube, Rp200 airway resistance and high PEEP with a low fresh gas flow). Exempting these, the calculated maximum inflation rate was above 60 /min in all experiments with Crs of 0.5 and 1.1 mL/ cm H_2_O (Figures 2, 3 and Supplementary Tables 2b, 3b). At higher compliances, 2.2 and 3.4 mL/cm H_2_O, the time constants were longer (>0.17 s) with a calculated safe maximum ventilatory rate below 60 for most TPR settings and in all experiments with ET-tubes.

### Resistance From TPR

The TPR valve resistance was related to the set fresh gas flow and PEEP level. Increasing PEEP at lower fresh gas flows showed an increase in time constants. The increase related to increased PEEP was attenuated when using 15 L/min of fresh gas flows. The absolute difference when increasing PEEP from 8 and 10 cm H_2_O was small (<0.06 s) (Figure 2 and Supplementary Table 2b).

The time constants were dependent on fresh gas flow. For example, at PEEP 5 cm H_2_O and 10 L/min, the expiratory time constant was 0.21 s (with Rp50 and compliance 2.2 mL/cm H_2_O). Increasing PEEP to 10 cm by adjusting the TPR resistance valve increased the time constant to 0.30 s. Increasing PEEP to 10 cm H_2_O by increasing the fresh gas flow reduced the time constant to 0.15 s.

### Airway and Endotracheal Resistance

The differences in time constants between no added resistance and the Rp50 airway resistance were small (<0.05 s). The higher resistance in the Rp200 airway resistance increased time constants, more pronounced with higher compliances (Figure 2 and Supplementary Table 2a).

The addition of ET-tubes at higher compliances, 2.2 and 3.4 mL/cm H_2_O, resulted in time constants between 0.25 and 0.81 s (Figure 2 and Supplementary Table 2a).

## Discussion

The time needed for passive expiration through TPR systems was investigated in a neonatal mechanical lung model. The findings indicate that inadvertent PEEP is a potential risk and concern when using a TPR for ventilation of infants. The highest risk for incomplete expiration would be when providing PPV at a high rate, using a TPR system with a low fresh gas flow and a high PEEP on an infant with high lung compliance. A common real world example is that of a resuscitated term asphyxiated infant who is apneic, intubated and with normal lung compliance.

Our experiments indicate that, in infants with compliant lungs, complete expiration cannot be guaranteed at inflation rates over 50 inflations per minute. For these infants, the simulations indicate that there is a high risk for delivering inadvertent PEEP already at 60 infaltions per minute with standard TPR settings. For simulations with low compliances (0.5 and 1.1 mL/cm H_2_O), the time needed for expiration was short and the risk of generating inadvertent PEEP should lower than at higher compliances.

The increased time needed for expiration at higher compliances was a consequence of the larger tidal volume released through the TPR valve, airway and ET-tube resistance. The close link between compliance and resistance was expected and is consistent with the definition of expiratory time constants as being the product of compliance and resistance (9).

System resistance and time constants were directly affected by the TPR settings. The resistance increased with higher PEEPs or lower fresh gas flows. This is explained by the tightening of the PEEP valve when using low fresh gas flows or when increasing PEEP. The results clearly show that it is possible to shorten expiratory time constants by changing PEEP using the fresh gas flow rather than the expiratory valve (Figure 2). The expiratory resistance responsible for the increased TPR expiratory time constants also explains the increased imposed work of breathing seen when TPR systems are used for CPAP in models with spontaneous breathing (11).

### Technical Considerations and Limitations

The presented deflation data was based on measurements in a lung model with a single compartment, linear compliance and a fixed parabolic resistance. The results cannot be directly translated into clinical guidelines.

The mechanical lung model experiments were limited to four levels of compliance, three levels of PEEP, one level of driving pressure, three levels of fresh gas flow and one T-piece system. Some combinations are clinically unlikely and only included for completeness. Clinical use of higher driving pressures above the used 15 cm H_2_O are common and will increase the risk of incomplete exhalations by the increased tidal volumes needed to be expired. Leakage was not included as it would reduce the expiratory time constants.

The risk of inadvertent PEEP directly corresponds to the expiratory time constant and the time allowed for expiration. We have used this relation to estimate risk of inadvertent PEEP even if the used methodology does not allow recording of inadvertent PEEP, breath stacking or loss of tidal volumes due to elevated PEEP. The calculated maximum inflation rate was not the primary outcome but was included as a rough clinical estimate, presuming a fixed inflation time of 0.5 s. As we did not study inflation time constants or rise times, no predictions could be made on the actual minimum time for a complete ventilatory cycle in the model.

### Clinical Relevance

Finer et al. reported of 120 airway pressure recordings of TPR resuscitations in extremely low birth weight newborn infants 8 examples of serious “toxic” inadvertent PEEP with elevations over set PEEPs between 1.7 to 10.3 cmH_2_O (12). Only three of the eight infants were intubated. Other published data relates to intubated infants receiving mechanical ventilation. Simbruner investigated inadvertent PEEP in 10 neonates with a mean weight of 1.4 kg, compliance below 1 mL/cm H_2_O and ventilated with pressure control at a ventilatory rate of 30 (±3.5) /min (13). They found inadvertent PEEP >1 cm H_2_O in 19 of 29 measurements. Cruces et al. measured inadvertent PEEP in 16 larger infants (<1 year) with bronchiolitis and volume control ventilation. They report a median inadvertent PEEP of 1.5 (IQR 1–4.8) cm H_2_O at a median ventilatory rate of 28 (IQR 26–30) /min (14).

Our calculations indicate that incomplete expirations could be present, especially in larger infants with a high compliance. TPR systems are approved for use in infants up to 10 kg and with higher compliances, the use of a TPR with standard flows will be increasingly hazardous. There is very little data on the biomechanical performance of these systems after the newborn period in infants up to 10 kg in weight. Tracy et al. showed the potential for TPRs to exhibit inadvertent PEEP used in infant models with compliance values of 9 mL/cm H_2_O (3). The risk of incomplete expiration will depend on the ventilation rate and inspiratory to expiratory ratio (I:E ratio) determined by the operator. Inadvertent PEEP could be present in preterm infants with low compliances at higher ventilatory rates and possibly with a high airway resistance, even if our results indicate that this is less likely.

It is not clear if a prolonged expiratory time, inadvertent PEEP and air trapping is dangerous or even beneficial for some patients. Using incomplete expiration by increasing expiratory resistance and vantilatory rate to build functional residual capacity (FRC), has been suggested, as a way to physiologically maintain FRC in animals and humans (15). The safety of intentional using this strategy is not known (5). If a higher mean airway pressure is desired, it would be more predictable to increase PEEP. Higher present inflow rates to the Neopuff TPR of 15 LPM rather than 8 or 10 LPM may reduce the potential for inadvertent PEEP but this must be preset by the operator before use and cannot safely be adjusting during TPR use (16). Another potential concern with increasing the fresh gas flow is the increased peak flow during inspiration.

At birth the lung mechanics change dynamically and clinicians need to be aware that the TPR design has a high expiratory flow resistance which increases the total system expiratory time. Of particular concern is the delivery of surfactant to an intubated infant. The sudden change in compliance will increase the risk of inadvertent PEEP from increased compliance and tidal volumes if inflation rates and PEEP remain unchanged.

### Future Directions

The presented study generates several hypotheses concerning basic mechanical properties of passive expiration through TPR systems. These principles could be confirmed in mathematical models of expiration. Differences in resistance between different brands of TPR devices should also be easy to investigate in bench studies even if, theoretically, there should be similar findings.

Studies on the clinical importance of inadvertent PEEP, air trapping and lung injury are needed. Studies could also explore the alternative hypotheses that the reduced peak flow seen with a prolonged inspiratory rise time and the increased expiratory time constants are beneficial. Possible systems for comparisons are low resistance resuscitation systems or ventilators with adequate expiratory valves (4, 17). The expiratory time constants of these systems are not known and need to be determined.

*In-vivo* trials using flow monitoring during resuscitation should be able to provide adequate data on incomplete expirations in infants and animals. Such recordings are available but expiratory time constants have not been reported. They should also allow for some hypotheses on adaptive approaches during resuscitation as compliance increase.

## Conclusion

The risk of incomplete expiration and the development of inadvertent PEEP when using TPR for PPV increases with (1) higher ventilatory rate and shorter time for expiration, (2) larger tidal volumes as seen with a higher driving pressure or higher compliance, and (3) higher resistance. These three basic mechanical features could be important during T-piece resuscitation. The results indicate that the risk of inadvertent PEEP would be highest in larger infants with compliant lungs. The risk can be reduced by increasing the fresh gas flow and allowing sufficient time for expiration. The clinical risks related to TPR use and inadvertent PEEP should be possible to assess in animal and infant experiments or by reviewing already conducted trials.

## Data Availability Statement

The raw data supporting the conclusions of this article will be made available by the authors, without undue reservation.

## Author Contributions

TD, MF, and SD developed the concept. TD, MT, and MH conducted the measurements and analyzed the results. All authors actively contributed to writing and reviewing the manuscript.

## Conflict of Interest

The authors declare that the research was conducted in the absence of any commercial or financial relationships that could be construed as a potential conflict of interest.
