# Supplementary material for: Neonatal Resuscitation With T-Piece Systems: Risk of Inadvertent PEEP Related to Mechanical Properties
Source: Front Pediatr. 2021 Jun 7;9:663249. doi: 10.3389/fped.2021.663249 (PMC8215339; doi:10.3389/fped.2021.663249)
Supplement: Supplementary file 1 [file Data_Sheet_1.PDF]

All inflations and simulations combined

Model	n	VTi [mL]	VTe [mL]	Pinf [cm H2O]	VT/Pinf [mL/cm H2O]
0.5	240	8.24 (8.19-8.29)	8.13 (8.10-8.15)	15.31 (15.29-15.33)	0.54 (0.53-0.54)
1.1	240	17.42 (17.36-17.47)	17.31 (17.26-17.35)	15.25 (15.23-15.26)	1.14 (1.14-1.15)
2.2	240	34.33 (34.23-34.44)	34.14 (34.05-34.22)	15.27 (15.25-15.29)	2.25 (2.24-2.26)
3.4	240	52.54 (52.42-52.66)	52.12 (52.00-52.25)	15.25 (15.23-15.26)	3.45 (3.44-3.45)

Lung model tested without T-piece systems (10 inflations at three level of airway resistance)

Model	n	VTi [mL]	VTe [mL]	Pinf [cm H2O]	VT/Pinf [mL/cm H2O]
0.5	30	8.30 (8.18-8.41)	8.06 (8.03-8.09)	15.39 (15.34-15.45)	0.54 (0.53-0.55)
1.1	30	17.57 (17.47-17.67)	17.27 (17.18-17.36)	15.22 (15.20-15.25)	1.15 (1.15-1.16)
2.2	30	34.73 (34.57-34.89)	34.15 (33.95-34.34)	15.28 (15.22-15.33)	2.27 (2.26-2.29)
3.4	30	53.25 (52.96-53.54)	52.29 (51.85-52.73)	15.21 (15.16-15.26)	3.50 (3.48-3.52)

Experiements with endotracheal tubes (no tube, 2.5, 3.0, 3.5) and airway resistance (no resistor, Rp 50, Rp 200) for 10 inflations at 5 cm H2O PEEP

Model	n	VTi [mL]	VTe [mL]	Pinf [cm H2O]	VT/Pinf [mL/cm H2O]
0.5	120	8.48 (8.43-8.52)	8.28 (8.26-8.30)	15.22 (15.21-15.24)	0.56 (0.55-0.56)
1.1	120	17.67 (17.61-17.72)	17.55 (17.53-17.58)	15.2 (15.19-15.22)	1.16 (1.16-1.17)
2.2	120	34.90 (34.82-34.98)	34.63 (34.58-34.69)	15.24 (15.22-15.26)	2.29 (2.28-2.30)
3.4	120	53.12 (53.03-53.21)	52.79 (52.72-52.86)	15.28 (15.26-15.30)	3.48 (3.47-3.48)

Experiements with thre levels of fresh gas flow (8, 10, 15 L/min) and three levels of PEEP (5, 8, 10 cm H2O) for 10 inflations

Model	n	VTi [mL]	VTe [mL]	Pinf [cm H2O]	VT/Pinf [mL/cm H2O]
0.5	90	7.91 (7.84-7.97)	7.95 (7.90-7.99)	15.4 (15.36-15.43)	0.51 (0.51-0.52)
1.1	90	17.03 (16.97-17.09)	16.98 (16.93-17.04)	15.31 (15.28-15.34)	1.11 (1.11-1.12)
2.2	90	33.45 (33.37-33.53)	33.47 (33.37-33.58)	15.3 (15.26-15.34)	2.19 (2.18-2.19)
3.4	90	51.53 (51.43-51.62)	51.18 (51.03-51.34)	15.22 (15.19-15.24)	3.39 (3.38-3.39)

Supplement table 1a: Properties of the four adiabatic lung model bottles used. Inspiratory (VTi) and expiratory (VTe) tidal volumes calculated from measured flow. Compliance (VT/Pinf) calculated as delivered averaged delivered tidal volume devided by inflation pressure (Pinf). Means (95% CI).
