# Supplementary material for: Neonatal Resuscitation With T-Piece Systems: Risk of Inadvertent PEEP Related to Mechanical Properties
Source: Front Pediatr. 2021 Jun 7;9:663249. doi: 10.3389/fped.2021.663249 (PMC8215339; doi:10.3389/fped.2021.663249)
Supplement: Supplementary file 2 [file Data_Sheet_2.PDF]

| Model Crs | Resistor | Pinf [cm H2O] | Vte [mL] | τrs [s] | Max VR (Ti 0.5s) |
|---|---|---|---|---|---|
| 0.5 | No | 15.39 (15.26-15.51) | 8.04 (8.00-8.07) | 0.09 (0.09-0.09) | 77.50 (76.90-78.11) |
| | Rp50 | 15.42 (15.29-15.54) | 8.02 (7.95-8.08) | 0.09 (0.09-0.09) | 77.29 (76.64-77.94) |
| | Rp200 | 15.38 (15.33-15.42) | 8.12 (8.07-8.17) | 0.11 (0.11-0.11) | 72.59 (72.42-72.75) |
| 1.1 | No | 15.28 (15.24-15.32) | 17.21 (17.14-17.28) | 0.09 (0.09-0.09) | 77.17 (76.55-77.80) |
| | Rp50 | 15.18 (15.11-15.25) | 17.07 (16.94-17.19) | 0.09 (0.09-0.09) | 77.00 (76.69-77.30) |
| | Rp200 | 15.21 (15.18-15.23) | 17.54 (17.43-17.64) | 0.21 (0.21-0.21) | 53.49 (53.38-53.60) |
| 2.2 | No | 15.25 (15.21-15.29) | 33.57 (33.43-33.72) | 0.09 (0.09-0.09) | 78.71 (78.48-78.93) |
| | Rp50 | 15.38 (15.25-15.52) | 34.11 (33.98-34.25) | 0.11 (0.11-0.11) | 71.16 (71.06-71.26) |
| | Rp200 | 15.20 (15.15-15.25) | 34.75 (34.63-34.88) | 0.42 (0.42-0.42) | 34.13 (34.07-34.18) |
| 3.4 | No | 15.22 (15.10-15.34) | 50.97 (50.75-51.18) | 0.10 (0.10-0.10) | 76.11 (75.89-76.32) |
| | Rp50 | 15.17 (15.13-15.22) | 52.17 (52.04-52.31) | 0.16 (0.16-0.16) | 61.09 (61.00-61.18) |
| | Rp200 | 15.24 (15.14-15.34) | 53.75 (53.62-53.87) | 0.64 (0.64-0.64) | 24.80 (24.76-24.84) |

**Supplement table 1b: Lung model properties of exhalation with airway resistors without T-piece resusciators.** Simulations at PEEP 0 cm H2O and inflation pressures 15cm H2O. Inflation pressure (Pinf) measured and expiratory tidal volume (Vte) with expiratory time constants (**τ**rs) calculated from flow measurement. Calculated maximum ventilator rate that assure complete exhalation (Max VR) was calculated as three expiratory time constants after a fixed inflation time of 0.5 seconds. Means (95% CI) for 10 consecutive inflations.
