# Supplementary material for: Neonatal Resuscitation With T-Piece Systems: Risk of Inadvertent PEEP Related to Mechanical Properties
Source: Front Pediatr. 2021 Jun 7;9:663249. doi: 10.3389/fped.2021.663249 (PMC8215339; doi:10.3389/fped.2021.663249)
Supplement: Supplementary file 3 [file Data_Sheet_3.PDF]

		Driver Flow		
		8 L/min	10 L/min	15 L/min
**Model Crs**	**PEEP**	**τrs [s]**	**τrs [s]**	**τrs [s]**
0.5	5 cm	0.08 (0.08-0.08)	0.08 (0.08-0.08)	0.08 (0.08-0.08)
	8 cm	0.10 (0.09-0.10)	0.08 (0.08-0.09)	0.07 (0.07-0.07)[A]
	10 cm	0.10 (0.10-0.10)	0.09 (0.09-0.09)	0.07 (0.07-0.07)[A]
1.1	5 cm	0.13 (0.13-0.13)	0.11 (0.11-0.11)	0.09 (0.09-0.09)
	8 cm	0.17 (0.17-0.17)	0.14 (0.14-0.14)	0.09 (0.09-0.09)[B]
	10 cm	0.18 (0.17-0.18)	0.15 (0.15-0.15)	0.09 (0.09-0.09)[B]
2.2	5 cm	0.25 (0.25-0.25)	0.21 (0.21-0.21)	0.15 (0.14-0.15)
	8 cm	0.33 (0.33-0.33)[C]	0.27 (0.27-0.27)	0.17 (0.17-0.17)
	10 cm	0.33 (0.33-0.33)[C]	0.30 (0.30-0.30)	0.16 (0.16-0.17)
3.4	5 cm	0.37 (0.37-0.37)	0.32 (0.31-0.32)	0.22 (0.22-0.22)
	8 cm	0.48 (0.47-0.49)	0.40 (0.40-0.40)	0.26 (0.26-0.26)
	10 cm	0.51 (0.50-0.52)	0.46 (0.45-0.46)	0.26 (0.26-0.26)

Lung model without TPR
**τrs [s]**
0.09 (0.09-0.09)
0.09 (0.09-0.09)
0.11 (0.11-0.11)
0.16 (0.16-0.16)

Supplement table 2a: Effect of driver flow, PEEP level (cm H2O) and compliance on expiratory time constants (τrs). Simulations without endotracheal tubes and airway resistance (Rp50). Simulations at PEEP 5 cm H2O and inflation pressures 15cm H2O. Lung model reference with Rp50 and inflation pressure of 15 cm H2O included (right) to allow comparison. Means (95% CI) for 10 consecutive inflations. All comparisons of compliance (Crs), driver flow and PEEP statistically significant exempt A-C.

		Driver Flow		
		8 L/min	10 L/min	15 L/min
**Model Crs**	**PEEP**	Max VR (Ti 0.5s)	Max VR (Ti 0.5s)	Max VR (Ti 0.5s)
0.5	5 cm	79.9 (79.55-80.34)	81.1 (80.85-81.40)	81.8 (81.38-82.27)
	8 cm	76.3 (75.89-76.71)	79.5 (79.27-79.73)	83.3 (83.13-83.46)[A]
	10 cm	75.4 (75.03-75.67)	78.4 (78.23-78.58)	83.7 (83.43-83.87)[A]
1.1	5 cm	67.2 (67.10-67.30)	72.0 (71.89-72.04)	79.2 (78.96-79.35)
	8 cm	60.0 (59.75-60.27)	65.9 (65.84-65.96)	78.7 (78.46-78.85)[B]
	10 cm	58.3 (57.96-58.61)	63.1 (62.92-63.29)	78.9 (78.82-79.06)[B]
2.2	5 cm	47.9 (47.82-47.99)	53.0 (52.95-53.06)	64.1 (64.00-64.19)
	8 cm	40.5 (40.37-40.64)[C]	46.2 (46.13-46.27)	60.1 (59.91-60.21)
	10 cm	40.3 (40.03-40.56)[C]	42.9 (42.75-42.97)	60.3 (60.12-60.51)
3.4	5 cm	37.0 (36.94-37.09)	41.5 (41.47-41.53)	51.7 (51.64-51.84)
	8 cm	30.9 (30.43-31.35)	35.3 (35.23-35.40)	47.0 (46.89-47.14)
	10 cm	29.5 (29.25-29.83)	32.1 (32.03-32.23)	46.6 (46.41-46.80)

Lung model without TPR
Max VR (Ti 0.5s)
77.3 (76.64-77.94)
77.0 (76.69-77.30)
71.2 (71.06-71.26)
61.1 (61.00-61.18)

Supplement table 2b: Effect of driver flow, PEEP level (cm H2O) and compliance on calculated maximum ventilator rate that assure complete exhalation (Max VR). Complete exhalation defined as three expiratory time constants after a fixed inflation time of 0.5 seconds. Simulations without endotracheal tubes and airway resistance (Rp50). Simulations at PEEP 5 cm H2O and inflation pressures 15cm H2O. Lung model reference with Rp50 and inflation pressure of 15 cm H2O included (right) to allow comparison. Means (95% CI) for 10 consecutive inflations. All comparisons of compliance (Crs), driver flow and PEEP statistically significant exempt A-C.
