# Supplementary material for: Neonatal Resuscitation With T-Piece Systems: Risk of Inadvertent PEEP Related to Mechanical Properties
Source: Front Pediatr. 2021 Jun 7;9:663249. doi: 10.3389/fped.2021.663249 (PMC8215339; doi:10.3389/fped.2021.663249)
Supplement: Supplementary file 4 [file Data_Sheet_4.PDF]

		Endotracheal tube #2.5	Endotracheal tube #3.0	Endotracheal tube #3.5	Endotracheal tube No	Lung model without TPR
Model Crs	Resistor	τrs [s]	τrs [s]	τrs [s]	τrs [s]	τrs [s]
0.5	No	0.10 (0.10-0.10)	0.09 (0.09-0.09)A	0.08 (0.08-0.09)	0.08 (0.08-0.08)	0.09 (0.09-0.09)
	Rp50	0.10 (0.10-0.10)	0.09 (0.09-0.09)A	0.09 (0.09-0.09)	0.08 (0.08-0.08)	0.09 (0.09-0.09)
	Rp200	0.13 (0.13-0.13)	0.12 (0.12-0.13)	0.12 (0.12-0.12)	0.11 (0.11-0.11)	0.11 (0.11-0.11)
1.1	No	0.18 (0.18-0.18)	0.15 (0.15-0.15)	0.13 (0.13-0.13)	0.10 (0.10-0.11)	0.09 (0.09-0.09)
	Rp50	0.19 (0.19-0.19)	0.15 (0.15-0.15)	0.14 (0.13-0.14)	0.11 (0.11-0.11)	0.09 (0.09-0.09)
	Rp200	0.27 (0.27-0.27)	0.25 (0.25-0.25)	0.24 (0.24-0.24)	0.22 (0.22-0.22)	0.21 (0.21-0.21)
2.2	No	0.35 (0.35-0.35)	0.28 (0.28-0.29)	0.25 (0.25-0.25)	0.20 (0.19-0.20)	0.09 (0.09-0.09)
	Rp50	0.37 (0.37-0.37)	0.30 (0.30-0.30)	0.26 (0.26-0.26)	0.21 (0.21-0.21)	0.11 (0.11-0.11)
	Rp200	0.54 (0.53-0.54)	0.50 (0.49-0.50)	0.47 (0.47-0.47)	0.45 (0.45-0.45)	0.42 (0.42-0.42)
3.4	No	0.54 (0.54-0.54)	0.42 (0.42-0.42)	0.37 (0.37-0.37)	0.30 (0.29-0.30)	0.10 (0.10-0.10)
	Rp50	0.56 (0.55-0.56)	0.46 (0.46-0.46)	0.40 (0.40-0.40)	0.32 (0.32-0.32)	0.16 (0.16-0.16)
	Rp200	0.81 (0.81-0.81)	0.75 (0.75-0.76)	0.72 (0.72-0.72)	0.67 (0.67-0.68)	0.64 (0.64-0.64)

Supplement table 3a: Effect of airway resistor, endotracheal tube size and compliance on expiratory time constants (τrs). Simulations at 10 L/min fresh gas flow, PEEP 5 cm H2O and inflation pressures 15 cm H2O. Lung model reference with inflation pressure of 15 cm H2O included (right) to allow comparison. Means (95% CI) for 10 consecutive inflations. Means compared with all comparisons of compliance (Crs), endotracheal tube and resistors statistically significant exempt A.

		Endotracheal tube #2.5	Endotracheal tube #3.0	Endotracheal tube #3.5	Endotracheal tube No	Lung model without TPR
Model Crs	Resistor	Max VR (Ti 0.5s)	Max VR (Ti 0.5s)	Max VR (Ti 0.5s)	Max VR (Ti 0.5s)	Max VR (Ti 0.5s)
0.5	No	75.4 (75.23-75.55)	78.2 (77.41-78.90)A	79.7 (79.45-79.90)	81.9 (81.63-82.10)	77.50 (76.90-78.11)
	Rp50	74.2 (73.77-74.65)	77.6 (77.33-77.88)A	79.0 (78.80-79.21)	81.0 (80.66-81.42)	77.29 (76.64-77.94)
	Rp200	66.6 (66.45-66.76)	68.7 (68.31-69.02)	69.7 (69.57-69.90)	71.3 (71.13-71.47)	72.59 (72.42-72.75)
1.1	No	58.0 (57.88-58.04)	63.7 (63.65-63.79)	68.1 (67.88-68.26)	73.8 (73.62-73.92)	77.17 (76.55-77.80)
	Rp50	56.2 (56.09-56.30)	62.4 (62.34-62.48)	66.2 (66.16-66.32)	71.5 (71.36-71.56)	77.00 (76.69-77.30)
	Rp200	45.5 (45.39-45.59)	48.1 (47.98-48.14)	49.5 (49.36-49.59)	51.5 (51.40-51.64)	53.49 (53.38-53.60)
2.2	No	38.8 (38.64-38.85)	44.4 (44.27-44.45)	48.3 (48.28-48.40)	55.3 (55.20-55.36)	78.71 (78.48-78.93)
	Rp50	37.4 (37.29-37.44)	42.9 (42.78-42.94)	46.7 (46.59-46.79)	52.6 (52.56-52.66)	71.16 (71.06-71.26)
	Rp200	28.5 (28.41-28.59)	30.2 (30.10-30.26)	31.4 (31.35-31.51)	32.6 (32.44-32.66)	34.13 (34.07-34.18)
3.4	No	28.4 (28.38-28.48)	34.0 (34.02-34.07)	37.2 (37.08-37.29)	43.3 (43.23-43.34)	76.11 (75.89-76.32)
	Rp50	27.7 (27.61-27.75)	32.1 (32.00-32.11)	35.1 (35.02-35.14)	40.9 (40.81-40.99)	61.09 (61.00-61.18)
	Rp200	20.5 (20.43-20.56)	21.7 (21.68-21.79)	22.6 (22.52-22.61)	23.8 (23.75-23.87)	24.80 (24.76-24.84)

Supplement table 3b: Effect of airway resistors, endotracheal tube size and compliance on calculated maximum ventilator rate (max VR) that assure complete expiration. Complete exhalation defined as three expiratory time constants after a fixed inhalation time of 0.5 seconds. Simulations at 10 L/min fresh gas flow, PEEP 5 cm H2O and inflation pressures 15cm H2O. Lung model reference with inflation pressure of 15 cm H2O included (right) to allow comparison. Means (95% CI) for 10 consecutive inflations. All comparisons of compliance (Crs), endotracheal tube and resistors statistically significant exempt A.
